# Association between Circulating Vitamin D Level and Urolithiasis: A Systematic Review and Meta-Analysis

**DOI:** 10.3390/nu9030301

**Published:** 2017-03-18

**Authors:** Henglong Hu, Jiaqiao Zhang, Yuchao Lu, Zongbiao Zhang, Baolong Qin, Hongbin Gao, Yufeng Wang, Jianning Zhu, Qing Wang, Yunpeng Zhu, Yang Xun, Shaogang Wang

**Affiliations:** Department and Institute of Urology, Tongji Hospital, Tongji Medical College, Huazhong University of Science and Technology, No. 1095 Jiefang Avenue, Wuhan 430030, China; huhenglong@hust.edu.cn (H.H.); medzjq@163.com (J.Z.); luyuchao@hust.edu.cn (Y.L.); zzb070@126.com (Z.Z.); qinbaolong@hust.edu.cn (B.Q.); mhbgao@163.com (H.G.); yfwangtjm@163.com (Y.W.); zhu_jianning@163.com (J.Z.); wangqingtjm@hust.edu.cn (Q.W.); zhuyunpeng2016@163.com (Y.Z.); tjxyang1993@163.com (Y.X.)

**Keywords:** vitamin D, 1,25 dihydroxyvitamin D, 25 hydroxyvitamin D, urolithiasis, nephrolithiasis, systematic review, meta-analysis

## Abstract

Many studies compared the serum/plasma 1,25 dihydroxyvitamin D_3_ (1,25(OH)_2_D) and 25 hydroxyvitamin D_3_ (25(OH)D) between people with and without nephrolithiasis, and their results were conflicting. After systematically searching PubMed, Web of Science, The Cochrane Library, CNKI, and the Wanfang Database, we conducted a meta-analysis. Thirty-two observational studies involving 23,228 participants were included. Meta-analysis of these studies showed that of stone formers (SFs), calcium SFs had significantly higher concentrations of 1,25(OH)_2_D (weighted mean difference (WMD), 10.19 pg/mL; 95% confidence interval (CI), 4.31–16.07; *p* = 0.0007 and WMD, 11.28 pg/mL; 95% CI, 4.07–18.50; *p* = 0.002, respectively) than non-stone formers, while the levels of 25(OH)D (WMD, 0.88 ng/mL; 95% CI, −1.04–2.80; *p* = 0.37 and WMD, −0.63 ng/mL; 95% CI, −2.72–1.47; *p* = 0.56, respectively) are similar. Compared with controls and normocalciuria SFs, hypercalciuria SFs had increased circulating 1,25(OH)_2_D (WMD, 9.41 pg/mL; 95% CI, 0.15–18.67; *p* = 0.05 and WMD, 2.75 pg/mL; 95% CI, −0.20–5.69; *p* = 0.07, respectively) and markedly higher 25(OH)D (WMD, 5.02 ng/mL; 95% CI, 0.99–9.06; *p* = 0.01 and WMD, 5.02 ng/mL; 95% CI, 2.14–7.90; *p* = 0.0006, respectively). Normocalciuria SFs had elevated 1,25(OH)_2_D level (WMD, 6.85 pg/mL; 95% CI, −5.00–18.71; *p* = 0.26) and comparable 25(OH)D (WMD, 0.94 ng/mL; 95% CI, −3.55–5.43; *p* = 0.68). Sensitivity analysis generated similar results. Current evidence suggests that increased circulating 1,25(OH)_2_D is associated with urinary stones and a higher level of circulating 25(OH)D is significantly associated with hypercalciuria urolithiasis. Further studies are still needed to reconfirm and clarify the role of vitamin D in the pathogenesis of stones.

## 1. Introduction

Urolithiasis is a common and recurring disease. The lifetime risk of renal stone disease is about 11% for men and 7% for women, and it tends to increase with changes in diet and climate globally [1]. In addition to high prevalence, it also has a recurrence rate as high as 50% at five years and 80%–90% at 10 years, respectively [2]. Although no data are available for China, medical care costs for urolithiasis were estimated to be $3.79 billion spent in the United States in 2007, and it will increase to $4.57 billion by 2030 due to the growth of the population and prevalence [3]. In addition to the economic burden, stones may result in hydronephrosis and life-threatening conditions, such as pyonephrosis and end-stage renal failure [4,5]. Although many minimally-invasive treatment procedures, such as extracorporeal shockwave lithotripsy, percutaneous nephrolithotomy, and ureteroscopy, have emerged for the treatment of renal and ureteral stones, all of these methods focus on the stone itself rather than “stone disease” [6,7]. Therefore, further investigation and clarification of the pathogenesis of nephrolithiasis are high priorities for the development and improvement of medical therapy and prevention.

Calcium is the most frequent component of urinary calculi and is the major constituent of nearly 75% of stones [8]. Hypercalciuria is the most common abnormality identified in calcium stone formers (SFs) [8]. Common calcium stones may originate from Randall’s plaques which can serve as anchors for stone growth [9]. Randall’s plaques are composed of calcium and phosphorus [8,9]. Vitamin D, a necessary hormone and nutrient for human, is the key regulator of calcium and phosphorus metabolism. Therefore, its major circulating metabolite, 25-hydroxyvitamin D_3_ (25(OH)D), and the active form, 1,25 dihydroxyvitamin D_3_ (1,25(OH)_2_D), are thought to play an important role in stone formation. Many studies have investigated the association between serum/plasma vitamin D and nephrolithiasis, however, their results are not consistent [10,11]. Therefore, we performed this systematic review and meta-analysis to determine the difference of serum/plasma 1,25(OH)_2_D and 25(OH)D levels between SFs and non-stone formers (non-SFs). In addition, we compared the circulating vitamin D concentrations of hypercalciuria and normocalciuria SFs with that of controls.

## 2. Materials and Methods

### 2.1. Literature Search and Study Selection

The Preferred Reporting Items for Systematic Reviews and Meta-Analyses (PRISMA) statement was followed by our study [12]. PubMed, Web of Science, The Cochrane Library, CNKI, and the Wanfang Database were systematically searched to identify relevant studies reporting the relationship between nephrolithiasis and circulating vitamin D. The search was performed on 31 December 2016. The initial search process was designed to find all relevant published original articles without limitation by year or language. Detailed search terms were: (stone* OR calculi OR calculus OR urolithiasis OR nephrolithiasis) AND (Calcitriol OR Cholecalciferol OR “vitamin D*” OR “1,25-Dihydroxyvitamin D*” OR “1,25-dihydroxycholecalciferol” OR “1,25-(OH)_2_D_3_” OR “1,25(OH)_2_D_3_” OR “25-Hydroxyvitamin D” OR 25-hydroxycholecalciferol OR 25-(OH)D_3_ OR 25(OH)D_3_). Two authors (Henglong Hu and Jiaqiao Zhang) independently screened all of the citations returned from the search strategy to identify potentially eligible studies. Studies comparing the circulating vitamin D between SFs and healthy controls or between different type of SFs were screened further. Conference abstracts were not included, as they were deemed methodologically inappropriate. Disagreements were resolved through discussions. If disagreement persists, a third investigator (Shaogang Wang) will be consulted to attain consensus.

### 2.2. Data Extraction and Study Quality Assessment

The following information from each eligible study will be extracted and entered into a pre-designed data extraction form by two investigators (Yunpeng Zhu and Yang Xun) independently: publication year and journal, authors, countries, study design, study period, sample size, participants’ characteristics (age, gender), vitamin D types, measurement methods, means, and standard deviations of 1,25(OH)_2_D and/or 25(OH)D, and the units. Disagreements between the two authors will be resolved by rechecking the article and discussion. If disagreement persists, a third investigator (Shaogang Wang) will be consulted to attain consensus. The methodological quality of each study was evaluated by the two authors mentioned above using the Newcastle-Ottawa Scale for non-randomized controlled trials [13]. Possible publication bias was assessed using funnel plots of the outcome comparisons.

### 2.3. Data Processing and Statistical Analysis

To reduce heterogeneity and make it easier to describe and understand, serum/plasma 1,25(OH)_2_D levels provided not in pg/mL were converted to that and serum/plasma 25(OH)D levels provided in other units of measurement were converted to ng/mL. If the standard error of the mean rather than standard deviation is provided, then the standard deviation would be calculated by multiplying the standard error of the mean by the square of sample size. Combined means or standard deviations were calculated following the method described in the Cochrane Handbook [14]. For studies presenting continuous data as means and range, standard deviations were calculated using the methodology proposed by Hozo et al. as previously described [15,16,17]. The meta-analysis was performed using Review Manager Software V.5.3 (RevMan V.5.3, The Cochrane Collaboration, Oxford, UK). The weighted mean difference (WMD) with 95% confidence intervals (CIs) were used as the summary statistics for continuous variables. Heterogeneity among studies was evaluated by chi-square test and *I*^2^ statistics. Moreover, the pooled estimates were calculated with the fixed-effect model if no significant heterogeneity was detected; otherwise, the random-effect model was used. The pooled effects were determined by the *z* test. A *p*-value less than 0.05 was considered statistically significant. Funnel plots assessing publication bias were generated using RevMan v.5.3. Additionally, a sensitivity analysis was performed by pooling only studies with a relatively high score (scored 8 and 9) of Newcastle-Ottawa Scale.

## 3. Results

### 3.1. Literature Search and Study Selection

**Figure 1:**
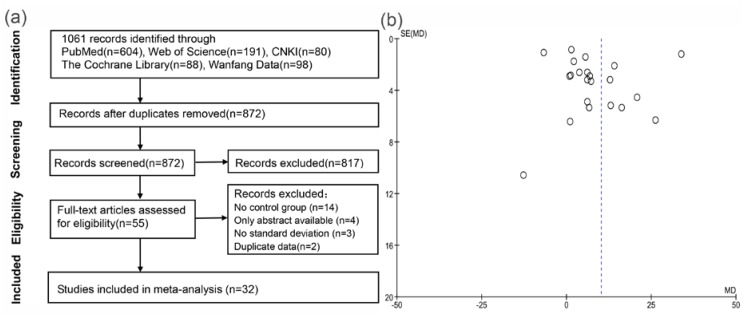
(**a**) Flowchart of the studies selection process; and (**b**) funnel plots for the difference of circulating 1,25(OH)D levels between SFs and control subjects.

The literature search and study selection process are depicted in Figure 1a. Electronic searches revealed 1061 articles. After screening of titles and abstracts, we considered 55 that were relevant to our purpose and, therefore, we retrieved the full-text articles, but four studies had only abstracts available. After full-text analysis, another 19 studies were excluded for the following reasons: 14 had no control group and no useful data, three did not provide standard deviations, and two reported duplicate data. Finally, 32 studies fulfilled our eligibility criteria and were enrolled in the meta-analysis [10,11,18,19,20,21,22,23,24,25,26,27,28,29,30,31,32,33,34,35,36,37,38,39,40,41,42,43,44,45,46,47].

### 3.2. Systematic Reviews of Included Studies

Table 1 summarizes characteristics of the eligible studies published from 1977 to 2016. Seven of the studies were conducted in the USA, three in Canada, three in Italy, two in France, two in Iran, two in Spain, two in Sweden, two in Turkey, and one each in Australia, Belgium, Brazil, Germany, Japan, Korea, Netherlands, Pakistan, and Switzerland. All studies were observational and two were cohort studies [44,46]. Most studies clearly demonstrated that they ruled out hyperparathyroidism and/or renal tubule acidosis [10,11,18,19,22,26,27,29,33,34,36,43], some also excluded patients using calcium and/or vitamin D [18,26,29,35,39,42]. Seventeen studies described their criteria of hypercalciuria and they were not consistent [18,19,20,22,24,26,28,29,32,35,36,37,38,42,43,45], twelve of which defined hypercalciuria as urinary calcium excretion of more than 300 mg/24 h for men and 250 mg for women or 4 mg/kg/day [19,23,26,28,29,32,35,36,38,42,43]. The subjects were on a free or normal calcium diet in some studies [10,11,19,21,22,26,28,29,32,35,36,39,43], while restricted calcium diets were adopted in some others [24,27,31,33,34,38]. Nineteen of the included trials only tested serum/plasma 1,25(OH)_2_D [19,20,22,24,26,27,28,30,31,32,33,34,35,36,37,38,39,42,43], six only assessed 25(OH)D [18,25,40,41,44,47], and the others evaluated the two metabolites at the same time [10,11,21,22,29,45,46]. The most commonly used measurement method for 1,25(OH)_2_D and 25(OH)D was radioimmunoassay. Twenty studies were rated as being relatively high in quality according to the Newcastle-Ottawa Scale [18,19,21,23,24,27,28,29,30,33,35,37,38,39,40,42,43,45,46,47]. The other 12 were scored as 6 or 7, mainly due to some important baseline characteristics of the groups, such as age, sex, and/or use of vitamin D were not well matched or clearly reported [10,11,20,22,25,26,31,32,34,36,41,44]. Additionally, a sensitivity analysis was conducted in order to detect and rule out any potential bias associated with the effects of such studies on the results as a whole. We also analyzed the possible publication bias by generating funnel plots of all of the evaluated comparisons. As an example, Figure 1b represents the funnel plot of the comparison of 1,25(OH)_2_D between stone and control group. As well as the other nine funnel plots presented in Figure S1, it does not show an obvious asymmetry; in other words, suggesting that publication bias was not significant (Figure 1b).

### 3.3. Meta-Analysis Results

**Figure 2:**
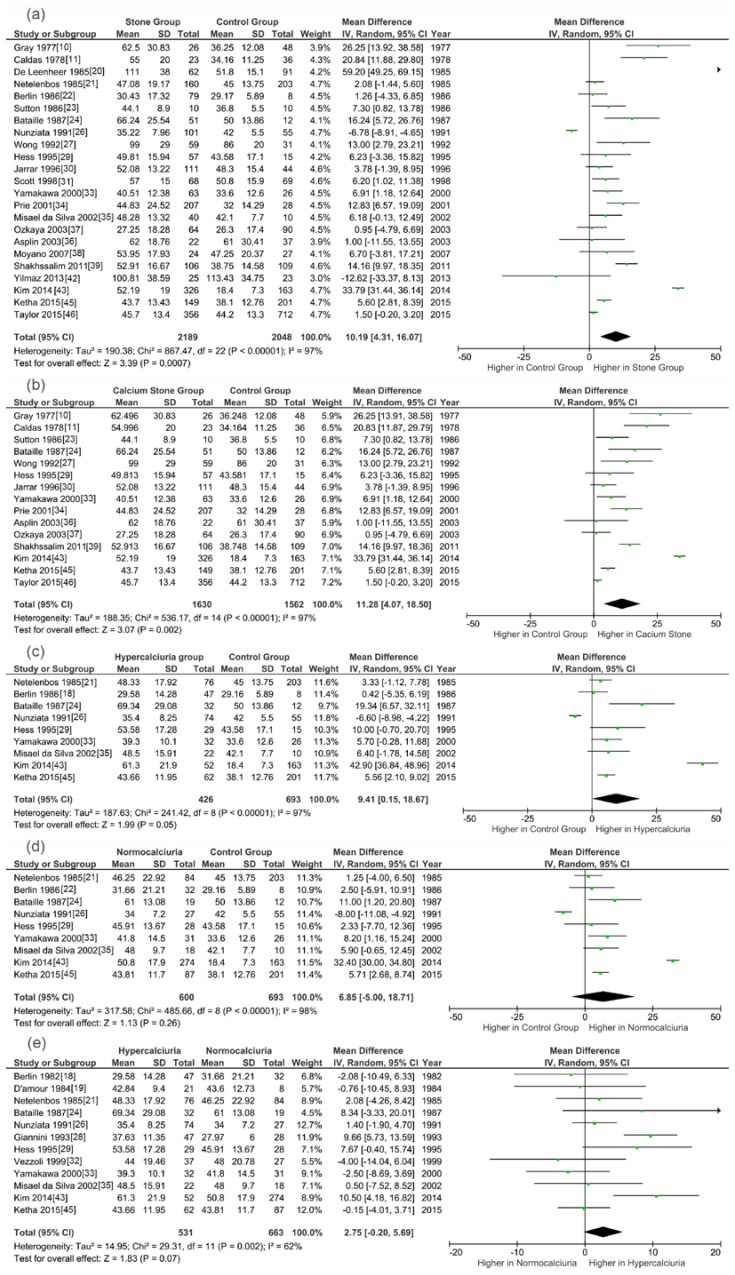
Forests plots of comparisons of circulating 1,25(OH)_2_D between different groups: (**a**) stone formers versus controls; (**b**) calcium stone formers versus controls; (**c**) hypercalciuria stone formers versus controls; (**d**) normocalciuria stone formers versus controls; and (**e**) hypercalciuria stone formers versus normocalciuria stone formers.

Pooling the data from 23 studies that assessed serum/plasma 1,25(OH)_2_D status revealed SFs had a significantly higher level of 1,25(OH)_2_D (WMD, 10.19 pg/mL; 95% CI, 4.31–16.07; *p* = 0.0007; Figure 2a) than controls. Further analysis demonstrated that, compared to non-SFs, calcium stone patients and hypercalciuria stone patients had increased concentrations of 1,25(OH)_2_D (WMD, 11.28 pg/mL; 95% CI, 4.07–18.50; *p* = 0.002 and WMD, 9.41 pg/mL; 95% CI, 0.15–18.67; *p* = 0.05, respectively). The circulating 1,25(OH)_2_D in hypercalciuria SFs tended to be higher than that of normocalciuria ones, while not reaching significance (WMD, 2.75 pg/mL; 95% CI, −0.20–5.69; *p* = 0.07). Normocalciuria SFs had elevated 1,25(OH)_2_D levels than the control subjects, but the difference was not significant (WMD, 6.85 pg/mL; 95% CI, −5.00–18.71; *p* = 0.26).

**Figure 3:**
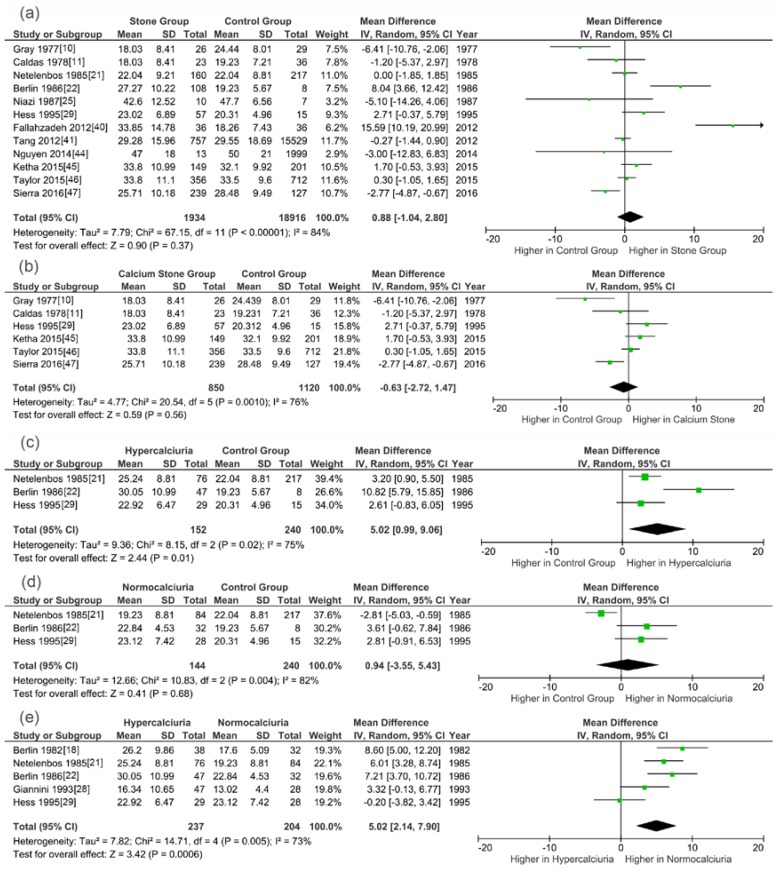
Forests plots of comparisons of circulating 25(OH)D between different groups: (**a**) stone formers versus controls; (**b**) calcium stone formers versus controls; (**c**) hypercalciuria stone formers versus controls; (**d**) normocalciuria stone formers versus controls; and (**e**) hypercalciuria stone formers versus normocalciuria stone formers.

Figure 3 shows the meta-analysis results of studies evaluating 25(OH)D levels. Meta-analysis of these studies showed that stone patients, calcium stone patients and normocalciuria SFs had similar serum/plasma 25(OH)D concentration with controls (WMD, 0.88 ng/mL, 95% CI, −1.04–2.80, *p* = 0.37; WMD, −0.63 ng/mL, 95% CI, −2.72–1.47, *p* = 0.56; WMD, 0.94 ng/mL, 95% CI, −3.55–5.43, *p* = 0.68, respectively). However, compared with non-SFs and normocalciuria stone patients, hypercalciuria SFs had markedly higher level of circulating 25(OH)D (WMD, 5.02 ng/mL; 95% CI, 0.99–9.06; *p* = 0.01 and WMD, 5.02 ng/mL; 95% CI, 2.14–7.90; *p* = 0.0006, respectively).

### 3.4. Sensitivity Analysis

Further sensitivity analysis was performed by removing studies scoring lower than 8 according to Newcastle-Ottawa Scale. As showed in Table 2, sensitivity analyses generated comparable results and suggested that the results of this meta-analysis were relatively stable and reliable. It is notable that the circulating 1,25(OH)_2_D of hypercalciuria SFs becomes significantly higher than that of the controls.

## 4. Discussion

Urolithiasis, an old disease, continues to be a major cause of kidney function loss and creates a significant burden on our public health system. Although many medical or surgical treatment methods have been developed, a necessary step to improve preventive and treatment outcomes is better understanding of the etiology and pathogenesis. Many systemic diseases, such as primary hyperparathyroidism, bowel disease and renal tubular acidosis, can cause the formation of calcium stones, but the majority of SFs are found to be with no systemic illness and they are called idiopathic SFs [48]. This study was mainly focused on these patients. 

Through a comprehensive meta-analysis, our results demonstrate that, compared to control subjects, urinary stone patients and calcium SFs have significantly higher 1,25(OH)_2_D concentrations while having similar 25(OH)D serum/plasma values. Although 25(OH)D is the major vitamin D circulating metabolite, 1,25(OH)_2_D is known to be the most important metabolite in regulating calcium and phosphorus metabolism and bone resorption. Calcium regulates a wide range of biological processes and the main constitution of bone. These findings suggest that 1,25(OH)_2_D might be an important intrinsic factor in stones, especially calcium stone formation.

Many idiopathic SFs have metabolic abnormalities that can be detected by 24 h urinalysis, but are not considered to be systemic diseases [48]. Hypercalciuria is the most common metabolic abnormality in patients with urolithiasis and the principal correlates of Randall’s plaque coverage [8,48,49]. One prerequisite for calcium oxalate overgrowth on Randall’s plaque is calcium oxalate supersaturation, which is also strongly linked to hypercalciuria. Subgroup meta-analyses were implemented to further clarify the potential relationship between hypercalciuria and vitamin D. Strikingly, the results demonstrated elevated circulating 1,25(OH)_2_D and 25(OH)D levels in hypercalciuria SFs than controls and normocalciuria SFs. This can be partly explained by the role of vitamin D in promoting intestinal absorption of calcium and bone reabsorption. Further analysis also found that 1,25(OH)_2_D levels were higher in normocalciuria stone patients than controls, but failed to reach significance (WMD, 6.85 pg/mL; 95% CI, −5.00–18.71, *p* = 0.26; *p* = 0.12 in the sensitivity analysis). The *p* value was relatively small and it further decreased in the sensitivity analysis, which suggests that the association between increased 1,25(OH)_2_D and normocalciuria stones may not be as tight as between higher 1,25(OH)_2_D concentrations and hypercalciuria stones. However, this still needs additional large volume studies to confirm. Similarly, normocalciuria SFs had comparable serum/plasma concentration of 25(OH)D with controls (WMD, 0.94 ng/mL, 95% CI, −3.55–5.43, *p* = 0.68; *p* = 0.94 in the sensitivity analysis).

Urolithiasis is a multifactorial disease and both genetic and environmental factors have effects on its onset and severity. Among these factors, the vitamin D signaling pathway plays an important role. Recent evidence has identified loss of function mutations in CYP24A1, encoding the vitamin D-24-hydroxylase which regulates the catabolism of 1,25(OH)_2_D, can result in high circulating levels of 1,25(OH)_2_D, hypercalcemia, hypercalciuria, and nephrolithiasis in humans [50]. Our, and other team’s, studies using genetic hypercalciuric stone-forming rats showed that vitamin D could take part in the pathogenesis of urolithiasis [51,52,53]. Vitamin D receptor knockdown in genetic hypercalciuric rats reduced calcium phosphate deposits in the kidneys [52]. A recent meta-analysis displayed a significant contribution of vitamin D receptor polymorphisms to urolithiasis risk [54]. Vitamin D receptor gene polymorphisms may influence vitamin D function, as well as its serum levels. In addition to the above, supplementation of vitamin D in humans and rats can induce hypercalciuria, renal calcification, and/or renal stones [55,56]. 

A recent meta-analysis evaluating the association between serum vitamin D levels and the risk of kidney stone was performed by Wang et al. [57]. Although the meta-analysis also showed that serum vitamin D levels in kidney stone patients were significantly higher than that in non-kidney stone controls, several limitations or mistakes existed, and these would make the results unconvincing. First, and the most importantly, two included studies only consisted of stone patients and some stone patients were mistakenly regarded as healthy controls. Second, the meta-analysis pooled 1,25(OH)_2_D and 25(OH)D indiscriminately. This seems inappropriate and would increase the bias. Third, this meta-analysis only included seven articles involving 451 kidney stone cases and 482 controls, which is much less than ours. Moreover, some values in the analysis were not accurate and, on some occasions, the standard error was taken as standard deviation.

The inclusion of large number of cases in this meta-analysis provided us with sufficient power to detect the association between increased circulating 1,25(OH)_2_D and urolithiasis. The results also provide some implications for the research of vitamin D supplementation. Although Reid and colleagues’ study challenged the role of vitamin D supplementation in improving bone mineral density [58,59], supplementation of vitamin D has many beneficial effects [60,61,62,63]. However, many doctors and patients are concerned about whether vitamin D repletion will increase the risk of urolithiasis, especially in SFs [64]. A recent systematic review and meta-analysis demonstrated that long-term vitamin D supplementation resulted in increased risks of hypercalcemia and hypercalciuria, but did not increase risk of kidney stones [55]. A randomized controlled trial conducted in 21 SFs found that high-dose and low-dose vitamin D supplementation had no effect on urine calcium excretion or the supersaturation of calcium salts [65]. However, the results still need further confirmation by large randomized controlled trials.

However, several limitations also exist in our analysis. First, the number of recruited patients in some studies was relatively small. However, the complete analysis of 32 different studies and the stable results from sensitivity analysis strengthen our conclusion. Second, meta-analyses of cross-sectional studies cannot be used for establishing a causative link, but this study provides reliable evidence for the association between 1,25(OH)_2_D and nephrolithiasis. Third, the heterogeneity of the included studies is high and it may be due to the differences in ethnicity, participant ages, measurement methods, measurement seasons, vitamin D supplementations, and hypercalciuria definitions. We used the random model to minimize the effect. Finally, we are unable to use these results to suggest specific pathogenesis and treatment strategies due to limited information.

## 5. Conclusions

The current evidence demonstrated that, compared to control subjects, patients with urinary stone, calcium stones had significantly higher levels of 1,25(OH)_2_D, while having similar concentration of 25(OH)D. The circulating concentrations of 1,25(OH)_2_D and 25(OH)D were higher in hypercalciuria stone patients than controls and normocalciuria stone patients. Normocalciuria stone patients and controls have comparable levels of 25(OH)D. These results suggest that increased circulating 1,25(OH)_2_D is associated with urinary stones and a higher level of circulating 25(OH)D is significantly associated with hypercalciuria urolithiasis. Further studies are still needed to reconfirm and clarify the role of vitamin D in the pathogenesis of stones, thus, bringing about new approaches for prevention and treatment of this disease.

## Figures and Tables

**Table 1:** Characteristics of included studies.

Surname of First Author	Year	Country	NOS Score	Sample Type	Measurement Method for 1,25(OH)_2_D	Measurement Method For 25(OH)D	Stone Component	Groups	Participant Number	Age (Mean ± SD)	Sex Ratio (M/F)
Gray [10]	1977	USA	7/9	plasma	Chromatin binding assay	competitive protein binding assay	calcium oxalate/apatite	SG	26	48	24/2
								CG	48	25	27/21
Caldas [11]	1978	USA	7/9	plasma	Cytosol binding assay	competitive protein binding assay	calcium oxalate/apatite	SG	23	NA	20/3
								CG	36	NA	22/14
Berlin [18]	1982	Sweden	8/9	serum	-	Isotope dilution-mass spectrometry	calcium oxalate/phosphate	HSG	38	NA	16/6
								NSG	32	NA	34/4
D’Amour [19]	1984	Canada	7/9	serum	Competitive binding assay	-	NA	HSG	21	36.16 ± 31.39	17/4
								NSG	8	31.8 ± 18.38	3/5
De Leenheer [20]	1985	Belgium	7/9	serum	Radioimmunoassay	-	NA	SG	62	NA	NA
								CG	91	NA	NA
Netelenbos [21]	1985	Netherlands	8/9	serum	Competitive protein binding assay	Competitive protein binding assay	NA	SG	160	43 ± 14	106/54
								CG	203	39 ± 11	147/70
Berlin [22]	1986	Sweden	8/9	serum	Radioreceptor assay	Isotope dilution-mass spectrometry	NA	SG	79	43 ± 3.33	NA
								CG	8	31 ± 6.83	NA
Sutton [23]	1986	Canada	9/9	serum	Cytosol receptor assay	-	Calcium	SG	10	47 ± 11	10/0
								CG	10	47 ± 10	10/0
Bataille [24]	1987	France	8/9	plasma	Radioimmunoassay	-	Calcium	SG	51	NA	29/22
								CG	12	NA	7/5
Niazi [25]	1987	Parkistan	7/9	serum	-	NA	NA	SG	10	34	NA
								CG	7	26	NA
Nunziata [26]	1991	Italy	7/9	serum	Competitive binding assay	-	NA	SG	101	NA	NA
								CG	55	NA	NA
Wong [27]	1992	Australia	8/9	serum	Microassay	-	Calcium	SG	59	46.59 ± 13.92	51/8
								CG	31	43.52 ± 13.55	20/11
Giannini [28]	1993	Italy	8/9	serum	Competitive protein binding assay	-	Calcium	HSG	47	40.5 ± 2.8	NA
								NSG	28	48.8 ± 2.6	NA
Hess [29]	1995	Switzerland	8/9	serum	Radioimmunoassay	Radioimmunoassay	Calcium	SG	57	NA	NA
								CG	15	NA	NA
Jarrar [30]	1996	Germany	9/9	serum	Radioreceptor assay	-	Calcium	SG	111	54.92 ± 23.36	64/47
								CG	44	53.34 ± 18.66	22/22
Scott [31]	1998	Canada	7/9	serum	Radioimmunoassay	-	Mixed	SG	68	NA	45/23
								CG	69	NA	26/43
Vezzoli [32]	1999	Italy	7/9	plasm	Radioreceptor assay	-	Calcium oxalate	HSG	37	NA	NA
								NSG	27	NA	NA
Yamakawa [33]	2000	Japan	9/9	serum	Radioreceptor assay	-	Calcium	SG	63	55.7 ± 12.5	47/16
								CG	26	55.9 ± 15.9	21/5
Prie [34]	2001	France	7/9	serum	Radioimmunoassay	-	Calcium	HSG	207	NA	NA
								NSG	28	NA	NA
Misael da Silva [35]	2002	Brazil	9/9	serum	Radioisotopic assay	-	NA	SG	40	34.77 ± 11.73	19/21
								CG	10	32.4 ± 8.4	5/5
Asplin [36]	2003	USA	7/9	serum	Radioreceptor assay	-	Calcium	SG	22	NA	15/7
								CG	37	NA	14/23
Ozkaya [37]	2003	Turkey	8/9	serum	Radioimmunoassay	-	Calcium	SG	64	6.7 ± 3.5	26/38
								CG	90	7.2 ± 2.3	47/43
Moyano [38]	2007	Spain	9/9	serum	Radioimmunoassay	-	NA	SG	24	45.5 ± 13.5	22/29
								CG	27	48.6 ± 15.4	9/12
Shakhssalim [39]	2011	Iran	9/9	serum	Enzyme Immunoassay	-	Calcium	SG	106	43.4 ± 6.9	106/0
								CG	109	38.4 ± 6.9	109/0
Fallahzadeh [40]	2012	Iran	9/9	serum	-	Electrochemiluminescence	NA	SG	36	0.7 ± 0.39	24/12
								CG	36	0.7 ± 0.39	22/14
Tang [41]	2012	USA	6/9	serum	-	Radioimmunoassay	NA	SG	757	54 ± 22.29	453/304
								CG	15529	43 ± 23.68	7175/8354
Yilmaz [42]	2013	Turkey	8/9	serum	Enzyme linked immunosorbent assay	-	NA	SG	25	8.08 ± 5.18	13/12
								CG	23	10.2 ± 3.64	11/12
Kim [43]	2014	Korea	9/9	serum	Radioimmunoassay	-	Calcium	SG	326	45.8 ± 12.3	204/122
								CG	163	NA	NA
Nguyen [44]	2014	USA	7/9	serum	-	Liquid chromatography and mass spectrometry	NA	SG	13	60 ± 10	8/5
								CG	1999	53 ± 14	767/1232
Ketha [45]	2015	USA	9/9	serum	Mass spectrometry	Mass spectrometry	Calcium	SG	149	NA	NA
								CG	201	NA	NA
Taylor [46]	2015	USA	9/9	plasma	Liquid chromatography–tandem mass spectrometry	Liquid chromatography–tandem mass spectrometry	Calcium	SG	356	57.4 ± 8.1	356/0
								CG	712	57.4 ± 8.1	712/0
Sierra [47]	2016	Spain	8/9	Serum	-	NA	Calcium	SG	239	49.61 ± 13.64	NA
								CG	127	52.09 ± 11.02	NA

NOS: Newcastle-Ottawa Scale; SD: standard difference; M/F: male/female; USA: United States of America; NA: not available; SG: stone group; CG: control group; HSG: hypercalciuria stone group; NSG: normocalciuria stone group.

**Table 2:** Sensitivity analysis of the meta-analysis.

Items	Comparisons	Sample Size	Tests for Heterogeneity		Analysis Model	Test for Overall Effect		WWD pg/mL or ng/mL	Higher in
			*I^2^*	p *		Z	p *	95% CI	
1,25(OH)_2_D	SG vs. CG	1601/1676	97%	<0.0001	Random	2.22	0.03	7.92 (0.93,14.91)	SG
	CSG vs. CG	1352/1413	98%	<0.0001	Random	2.26	0.02	9.94 (1.34,18.56)	CSG
	HSG vs. CG	305/630	95%	<0.0001	Random	2.39	0.02	13.21 (2.38,24.04)	HSG
	NSG vs. CG	541/630	98%	<0.0001	Random	1.55	0.12	9.67 (−2.55,21.89)	NSG
	HSG vs. NSG	420/609	65%	0.002	Random	1.88	0.06	3.39 (−0.13,6.91)	HSG
25(OH)D	SG vs. CG	997/1308	98%	<0.0001	Random	1.48	0.14	2.02 (−0.66,4.69)	SG
	CSG vs. CG	801/1055	75%	0.007	Random	0.31	0.75	0.33 (−1.76,2.43)	CSG
	HSG vs. CG	105/232	0%	0.78	Fixed	3.09	0.002	3.02 (1.10,4.93)	HSG
	NSG vs. CG	112/232	85%	0.01	Random	0.07	0.94	−0.21 (−5.70,5.29)	CG
	HSG vs. NSG	190/172	77%	0.005	Random	2.53	0.01	4.48 (1.01,7.95)	HSG

CI: confidence interval; WMD: weighted mean difference. SG: stone group; CG: control group; CSG: calcium stone group; HSG: hypercalciuria stone group; NSG: normocalciuria stone group.* *p* < 0.05 was considered statistically significant.

## Supplementary Materials

The following are available online at http://www.mdpi.com/2072-6643/9/3/301/s1, Figure S1: Funnel plots of comparisons.

## References

[1] Scales C.D., Smith A.C., Hanley J.M., Saigal C.S. (2012). Prevalence of kidney stones in the United States. Eur. Urol..

[2] Skolarikos A., Straub M., Knoll T., Sarica K., Seitz C., Petrik A., Turk C. (2015). Metabolic evaluation and recurrence prevention for urinary stone patients: Eau guidelines. Eur. Urol..

[3] Antonelli J.A., Maalouf N.M., Pearle M.S., Lotan Y. (2014). Use of the national health and nutrition examination survey to calculate the impact of obesity and diabetes on cost and prevalence of urolithiasis in 2030. Eur. Urol..

[4] Sigurjonsdottir V.K., Runolfsdottir H.L., Indridason O.S., Palsson R., Edvardsson V.O. (2015). Impact of nephrolithiasis on kidney function. BMC Nephrol..

[5] Alexander R.T., Hemmelgarn B.R., Wiebe N., Bello A., Morgan C., Samuel S., Klarenbach S.W., Curhan G.C., Tonelli M.A. (2012). Kidney stones and kidney function loss: A cohort study. Br. Med. J..

[6] Turk C., Petrik A., Sarica K., Seitz C., Skolarikos A., Straub M., Knoll T. (2016). Eau guidelines on interventional treatment for urolithiasis. Eur. Urol..

[7] Hu H., Lu Y., He D., Cui L., Zhang J., Zhao Z., Qin B., Wang Y., Lin F., Wang S. (2016). Comparison of minimally invasive percutaneous nephrolithotomy and flexible ureteroscopy for the treatment of intermediate proximal ureteral and renal stones in the elderly. Urolithiasis.

[8] Pearle M.S., Antonelli J.A., Lotan Y., Wein A.J., Kavoussi L.R., Partin A.W., Peters A.C. (2016). Urinary lithiasis: Etiology, epidemiology, and pathogenesis. Campbell-Walsh Urology.

[9] Daudon M., Bazin D., Letavernier E. (2015). Randall's plaque as the origin of calcium oxalate kidney stones. Urolithiasis.

[10] Gray R.W., Wilz D.R., Caldas A.E., Lemann J. (1977). The importance of phosphate in regulating plasma 1,25-(OH)2-vitamin D levels in humans: Studies in healthy subjects in calcium-stone formers and in patients with primary hyperparathyroidism. J. Clin. Endocrinol. Metab..

[11] Caldas A.E., Gray R.W., Lemann J. (1978). The simultaneous measurement of vitamin D metabolites in plasma: Studies in healthy adults and in patients with calcium nephrolithiasis. J. Lab. Clin. Med..

[12] Moher D., Liberati A., Tetzlaff J., Altman D.G., RISMA Group (2009). PPreferred reporting items for systematic reviews and meta-analyses: The prisma statement. PLoS Med..

[13] Wells G., Shea B., O’Connell D., Peterson J., Welch V., Losos M., Tugwell P. The Newcastle-Ottawa Scale (NOS) for Assessing the Quality of Nonrandomised Studies in Meta-Analyses. Http://www.Ohri.Ca/programs/clinical_epidemiology/oxford.Asp.

[14] Higgins J.P.T., Green S. Cochrane Handbook for Systematic Reviews of Interventions Version 5.1.0 (Updated March 2011). www.handbook.cochrane.org.

[15] Hozo S.P., Djulbegovic B., Hozo I. (2005). Estimating the mean and variance from the median, range, and the size of a sample. BMC Med. Res. Methodol..

[16] Hu H., Lu Y., Cui L., Zhang J., Zhao Z., Qin B., Wang Y., Wang Q., Wang S. (2016). Impact of previous open renal surgery on the outcomes of subsequent percutaneous nephrolithotomy: A meta-analysis. BMJ Open.

[17] Hu H., Qin B., He D., Lu Y., Zhao Z., Zhang J., Wang Y., Wang S. (2015). Regional versus general anesthesia for percutaneous nephrolithotomy: A meta-analysis. PLoS ONE.

[18] Berlin T., Bjorkhem I., Collste L., Holmberg I., Wijkstrom H. (1982). Relation between hypercalciuria and vitamin D3-status in patients with urolithiasis. Scand. J. Urol. Nephrol..

[19] D’Amour P., Gascon-Barre M., Dufresne L., Perreault J.P. (1984). Influence of dietary calcium on serum 1,25-dihydroxyvitamin D concentrations in renal stone formers. Clin. Endocrinol. (Oxf.).

[20] De Leenheer A.P., Bauwens R.M. (1985). Radioimmunoassay for 1,25-dihydroxyvitamin D in serum or plasma. Clin. Chem..

[21] Netelenbos J.C., Jongen M.J., van der Vijgh W.J., Lips P., van Ginkel F.C. (1985). Vitamin D status in urinary calcium stone formation. Arch. Intern. Med..

[22] Berlin T., Holmberg I., Bjorkhem I. (1986). High circulating levels of 25-hydroxyvitamin D3 in renal stone formers with hyperabsorptive hypercalciuria. Scand. J. Clin. Lab. Investig..

[23] Sutton R.A., Walker V.R. (1986). Bone resorption and hypercalciuria in calcium stoneformers. Metabolism.

[24] Bataille P., Bouillon R., Fournier A., Renaud H., Gueris J., Idrissi A. (1987). Increased plasma concentrations of total and free 1,25-(OH)2D3 in calcium stone formers with idiopathic hypercalciuria. Contrib. Nephrol..

[25] Niazi M.K., Khanum A., Sheikh M.A., Naqvi S.A. (1987). Study of 25-hydroxy vitamin D3, calcium, phosphorus in normal subjects and patients with calculi. J. Pak. Med. Assoc..

[26] Nunziata V., di Giovanni G., Giannattasio R., Lettera A.M., Mancini M. (1991). Recurrent kidney stones: Causes and diagnostic criteria in patients from Campania (Southern Italy). Br. J. Urol..

[27] Wong S.Y., Slater S.R., Evans R.A., Mason R., Lancaster E.K., Acland S.M., Eade Y., Hills E., Dunstan C.R. (1992). Metabolic studies in kidney stone disease. Q. J. Med..

[28] Giannini S., Nobile M., Castrignano R., Pati T., Tasca A., Villi G., Pellegrini F., D’Angelo A. (1993). Possible link between vitamin D and hyperoxaluria in patients with renal stone disease. Clin. Sci..

[29] Hess B., Ackermann D., Essig M., Takkinen R., Jaeger P. (1995). Renal mass and serum calcitriol in male idiopathic calcium renal stone formers: Role of protein intake. J. Clin. Endocrinol. Metab..

[30] Jarrar K., Amasheh R.A., Graef V., Weidner W. (1996). Relationship between 1,25-dihydroxyvitamin-D, calcium and uric acid in urinary stone formers. Urol. Int..

[31] Scott P., Ouimet D., Proulx Y., Trouve M.L., Guay G., Gagnon B., Valiquette L., Bonnardeaux A. (1998). The 1 alpha-hydroxylase locus is not linked to calcium stone formation or calciuric phenotypes in French-Canadian families. J. Am. Soc. Nephrol..

[32] Vezzoli G., Caumo A., Baragetti I., Zerbi S., Bellinzoni P., Centemero A., Rubinacci A., Moro G., Adamo D., Bianchi G. (1999). Study of calcium metabolism in idiopathic hypercalciuria by strontium oral load test. Clin. Chem..

[33] Yamakawa K., Kawamura J. (2000). Analysis of hypophosphatemia in calcium nephrolithiasis. Mol. Urol..

[34] Prie D., Ravery V., Boccon-Gibod L., Friedlander G. (2001). Frequency of renal phosphate leak among patients with calcium nephrolithiasis. Kidney Int..

[35] Misael da Silva A.M., dos Reis L.M., Pereira R.C., Futata E., Branco-Martins C.T., Noronha I.L., Wajchemberg B.L., Jorgetti V. (2002). Bone involvement in idiopathic hypercalciuria. Clin. Nephrol..

[36] Asplin J.R., Bauer K.A., Kinder J., Muller G., Coe B.J., Parks J.H., Coe F.L. (2003). Bone mineral density and urine calcium excretion among subjects with and without nephrolithiasis. Kidney Int..

[37] Ozkaya O., Soylemezoglu O., Misirlioglu M., Gonen S., Buyan N., Hasanoglu E. (2003). Polymorphisms in the vitamin d receptor gene and the risk of calcium nephrolithiasis in children. Eur. Urol..

[38] Moyano M.J., de Tejada M.J.G., Lozano R.G., Moruno R., Ortega R., Marti V., Palencia R.S., Miranda M.J., Palma A., Cano R.P. (2007). Alterations in bone mineral metabolism in patients with calcium kidney stone disease and polymorphism of vitamin D receptor. Preliminary results. Nefrologia.

[39] Shakhssalim N., Gilani K.R., Parvin M., Torbati P.M., Kashi A.H., Azadvari M., Golestan B., Basiri A. (2011). An assessment of parathyroid hormone, calcitonin, 1,25 (OH)(2) vitamin D3, estradiol and testosterone in men with active calcium stone disease and evaluation of its biochemical risk factors. Urol. Res..

[40] Fallahzadeh M.H., Zare J., Al-Hashemi G.H., Derakhshan A., Basiratnia M., Arasteh M.M., Fallahzadeh M.A., Fallahzadeh M.K. (2012). Elevated serum levels of vitamin D in infants with urolithiasis. Iran. J. Kidney Dis..

[41] Tang J., McFann K.K., Chonchol M.B. (2012). Association between serum 25-hydroxyvitamin D and nephrolithiasis: The national health and nutrition examination survey III, 1988–1994. Nephrol. Dial. Transplant..

[42] Yilmaz D., Sonmez F., Yenisey C., Girisgen I. (2013). The role of active vitamin D on stone formation and hypercalciuria. Nobel Med..

[43] Kim W.T., Kim Y.-J., Yun S.J., Shin K.-S., Choi Y.D., Lee S.C., Kim W.-J. (2014). Role of 1,25-dihydroxy vitamin D-3 and parathyroid hormone in urinary calcium excretion in calcium stone formers. Yonsei Med. J..

[44] Nguyen S., Baggerly L., French C., Heaney R.P., Gorham E.D., Garland C.F. (2014). 25-hydroxyvitamin D in the range of 20 to 100 ng/mL and incidence of kidney stones. Am. J. Public Health.

[45] Ketha H., Singh R.J., Grebe S.K., Bergstralh E.J., Rule A.D., Lieske J.C., Kumar R. (2015). Altered calcium and vitamin D homeostasis in first-time calcium kidney stone-formers. PLoS ONE.

[46] Taylor E.N., Hoofnagle A.N., Curhan G.C. (2015). Calcium and phosphorus regulatory hormones and risk of incident symptomatic kidney stones. Clin. J. Am. Soc. Nephrol..

[47] Sierra Giron-Prieto M., del Carmen Cano-Garcia M., Angel Arrabal-Polo M., Poyatos-Andujar A., Quesada-Charneco M., de Haro-Munoz T., Arias-Santiago S., Arrabal-Martin M. (2016). Analysis of vitamin D deficiency in calcium stone-forming patients. Int. Urol. Nephrol..

[48] Coe F.L., Worcester E.M., Evan A.P. (2016). Idiopathic hypercalciuria and formation of calcium renal stones. Nat. Rev. Nephrol..

[49] Kuo R.L., Lingeman J.E., Evan A.P., Paterson R.F., Parks J.H., Bledsoe S.B., Munch L.C., Coe F.L. (2003). Urine calcium and volume predict coverage of renal papilla by randall’s plaque. Kidney Int..

[50] Carpenter T.O. (2017). Cyp24a1 loss of function: Clinical phenotype of monoallelic and biallelic mutations. J. Steroid Biochem. Mol. Biol..

[51] Xi Q.L., Wang S.G., Ye Z.Q., Zhu Z.W., Li C., Bai J., Yu X., Liu J.H. (2011). Effect of silencing VDR gene in kidney on renal epithelial calcium transporter proteins and urinary calcium excretion in genetic hypercalciuric stone-forming rats. Urology.

[52] Jia Z., Wang S., Tang J., He D., Cui L., Liu Z., Guo B., Huang L., Lu Y., Hu H. (2014). Does crystal deposition in genetic hypercalciuric rat kidney tissue share similarities with bone formation?. Urology.

[53] Frick K.K., Krieger N.S., Bushinsky D.A. (2015). Modeling hypercalciuria in the genetic hypercalciuric stone-forming rat. Curr. Opin. Nephrol. Hypertens..

[54] Liu W., Chen M., Li M., Ma H., Tong S., Lei Y., Qi L. (2014). Vitamin d receptor gene (VDR) polymorphisms and the urolithiasis risk: An updated meta-analysis based on 20 case-control studies. Urolithiasis.

[55] Malihi Z., Wu Z., Stewart A.W., Lawes C.M.M., Scragg R. (2016). Hypercalcemia, hypercalciuria, and kidney stones in long-term studies of vitamin D supplementation: A systematic review and meta-analysis. Am. J. Clin. Nutr..

[56] Letavernier E., Verrier C., Goussard F., Perez J., Huguet L., Haymann J.-P., Baud L., Bazin D., Daudon M. (2016). Calcium and vitamin D have a synergistic role in a rat model of kidney stone disease. Kidney Int..

[57] Wang H., Man L., Li G., Huang G., Liu N. (2016). Association between serum vitamin D levels and the risk of kidney stone: Evidence from a meta-analysis. Nutr. J..

[58] Karras S.N., Anagnostis P., Beauchet O., Goulis D.G., Annweiler C. (2014). Vitamin D supplements and bone mineral density. Lancet.

[59] Reid I.R., Bolland M.J., Grey A. (2014). Effects of vitamin d supplements on bone mineral density: A systematic review and meta-analysis. Lancet.

[60] Grober U., Reichrath J., Holick M.F. (2015). Live longer with vitamin D?. Nutrients.

[61] Kim M.J., Kim S.N., Lee Y.W., Choe Y.B., Ahn K.J. (2016). Vitamin D status and efficacy of vitamin D supplementation in atopic dermatitis: A systematic review and meta-analysis. Nutrients.

[62] Pham T.M., Ekwaru J.P., Setayeshgar S., Veugelers P.J. (2015). The effect of changing serum 25-hydroxyvitamin D concentrations on metabolic syndrome: A longitudinal analysis of participants of a preventive health program. Nutrients.

[63] Shen L., Ji H.F. (2015). Associations between vitamin D status, supplementation, outdoor work and risk of parkinson's disease: A meta-analysis assessment. Nutrients.

[64] Johri N., Jaeger P., Ferraro P.M., Shavit L., Nair D., Robertson W.G., Gambaro G., Unwin R.J. (2016). Vitamin D deficiency is prevalent among idiopathic stone formers, but does correction pose any risk?. Urolithiasis.

[65] Ferroni M.C., Rycyna K.J., Averch T.D., Semins M.J. (2016). Vitamin D repletion in kidney stone formers: A randomized controlled trial. J. Urol..

