# Supplementary material for: Association between Circulating Vitamin D Level and Urolithiasis: A Systematic Review and Meta-Analysis
Source: Nutrients. 2017 Mar 18;9(3):301. doi: 10.3390/nu9030301 (PMC5372964; doi:10.3390/nu9030301)
Supplement: Supplementary file 1 [file nutrients-09-00301-s001.pdf]

Supplementary Materials: Association between circulating vitamin D Level and urolithiasis: a systematic review and meta-analysis

Henglong Hu, Jiaqiao Zhang, Yuchao Lu, Zongbiao Zhang, Baolong Qin, Hongbin Gao, Yufeng Wang, Jianning Zhu, Qing Wang, Yupeng Zhu, Yang Xun, Shaogang Wang

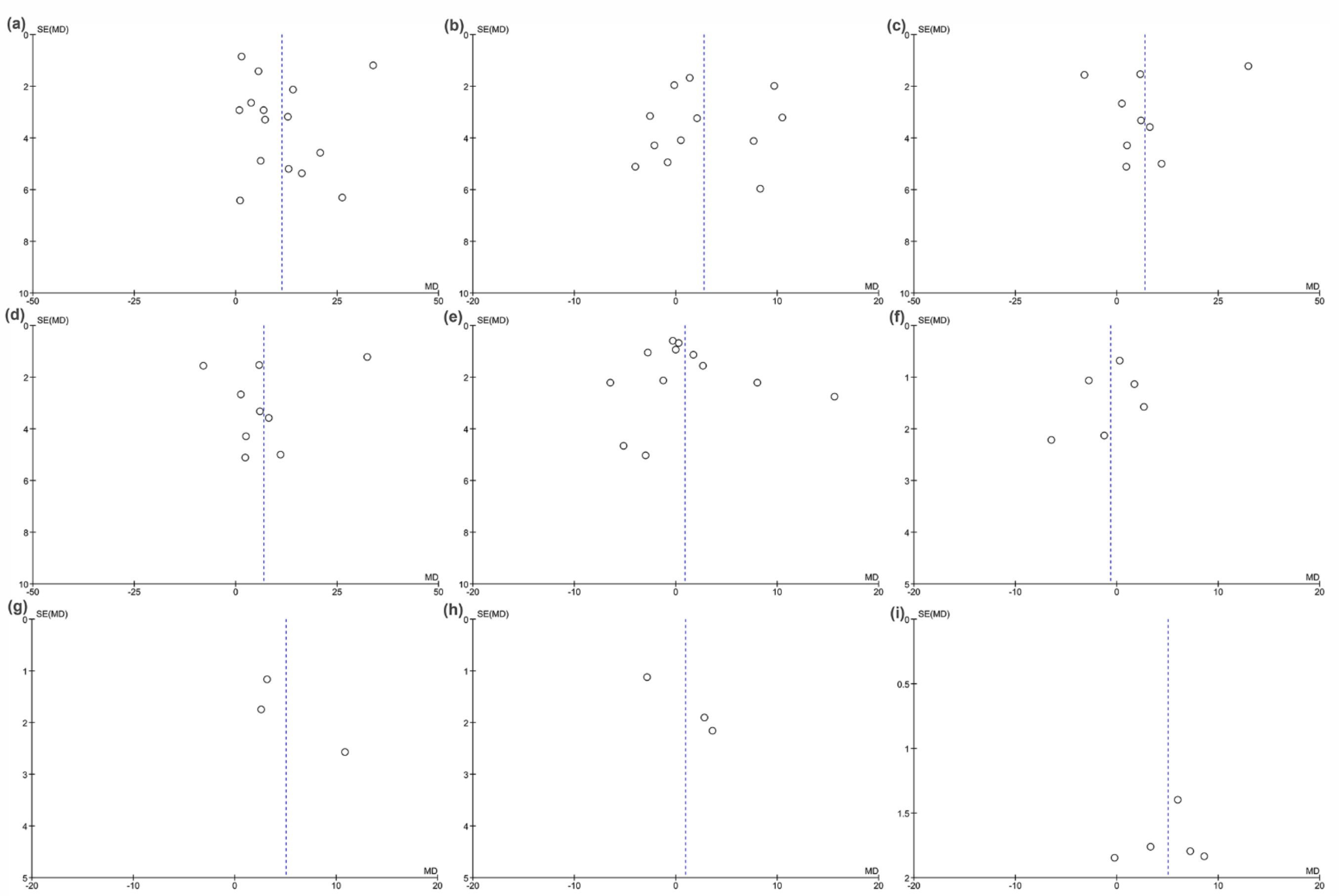

Figure S1. Funnel plots of comparisons of circulating 1,25(OH)2D between: (**a**) calcium stone formers and controls; (**b**) hypercalciuria stone formers and controls; (**c**) normocalciuria stone formers and controls; (**d**) hypercalciuria stone formers and normocalciuria stone formers. Funnel plots of comparisons of circulating 25(OH)D between: (**e**)stone formers and controls; (**f**) calcium stone formers and controls; (**g**) hypercalciuria stone formers and controls; (**h**) normocalciuria stone formers and controls; (**i**) hypercalciuria stone formers and normocalciuria stone formers.
